# Effect of Cation Type on the Isothermal Crystallization of Poly(vinylidene fluoride) Blended in Ionic Liquids with [Eu(tta)_4_]^−^ Anion

**DOI:** 10.1021/acs.jpcc.6c00153

**Published:** 2026-02-20

**Authors:** Luis A. Martins, José Luis Gómez Ribelles, Carlos M. Costa, Verónica de Zea Bermudez, Daniela M. Correia, Madalena Dionisio, Andreu Andrio, Ivan Krakowsky, Roser Sabater i Serra, Senentxu Lanceros-Méndez, Isabel Tort-Ausina

**Affiliations:** † Centre for Biomaterials and Tissue Engineering, CBIT, 16774 Universitat Politècnica de València, C/Camino de Vera s/n, 46022 Valencia, Spain; ‡ CIBER de Bioingeniería, Biomateriales y Nanomedicina, Instituto de Salud Carlos III, 28029 Madrid, Spain; § Physics Center of Minho and Porto Universities (CF-UM-UP) and Laboratory of Physics for Materials and Emergent Technologies, LapMET, 56059 University of Minho, 4710-057 Braga, Portugal; ∥ Institute of Science and Innovation for Bio-Sustainability (IB-S), University of Minho, 4710-053 Braga, Portugal; ⊥ Chemistry Department and CQ-VR, 56066 University of Trás-os-Montes e Alto Douro, 5000-801 Vila Real, Portugal; # Centre of Chemistry, University of Minho, 4710-057 Braga, Portugal; ∇ LAQV-REQUIMTE, Department of Chemistry, NOVA School of Science and Technology, 119482 Universidade Nova de Lisboa, 2829-516 Caparica, Portugal; ○ Departament de Física, 16748 Universitat Jaume I, 12071 Castelló, Spain; ◆ Department of Macromolecular Physics, Charles University, 180 00 Prague 8, Czech Republic; ¶ BCMaterials, Basque Center for Materials, Applications and Nanostructures, UPV/EHU Science Park, 48940 Leioa, Spain; ⋈ Ikerbasque, Basque Foundation for Science, 48009 Bilbao, Spain

## Abstract

To develop smart materials with tailored functional response, the combination of poly­(vinylidene fluoride) (PVDF) and advanced ionic additives such as ionic liquids (ILs) is increasingly being investigated. Depending on the processing conditions, the incorporation of these additives into PVDF, together with their functional response, promotes the nucleation of specific electroactive phases. This work explores the effect of incorporating sodium tetra­(2-thenoyltrifluoroacetonate) europate­(III), Na­[Eu­(tta)_4_] and 1-butyl-3-methylimidazolium tetra­(2-thenoyltrifluoroacetonate) europate­(III), [Bmim]­[Eu­(tta)_4_], into PVDF matrices through a comprehensive analysis of isothermal crystallization behavior, morphological features, crystalline phase development, and dielectric behavior. Field-emission scanning electron microscopy (FESEM) was used to analyze the microstructure, while Fourier transform infrared (FTIR) spectroscopy was used to assess the development of PVDF crystalline phases during its isothermal crystallization at various temperatures. All samples exhibited α, β, and γ crystalline phases, although their relative proportions differed significantly depending on the type of filler used. This suggests that [Bmim]­[Eu­(tta)_4_] is a strong promoter of the electroactive (EA) phases of PVDF. The results are attributed to the interaction between the IL charges and the PVDF dipoles of the EA structures, which are promoted by higher crystallization temperatures, as supported by both FTIR and DSC data. Thus, the addition of Na­[Eu­(tta)_4_] and [Bmim]­[Eu­(tta)_4_] strongly influences the crystallization kinetics of PVDF and allows nucleation of specific phases of PVDF. Additionally, dielectric spectroscopy revealed that the nature of the cation strongly influences conductivity behavior, as demonstrated by the dielectric results. Overall, the incorporation of Na­[Eu­(tta)_4_] and [Bmim]­[Eu­(tta)_4_] not only influences the crystallization kinetics of PVDF but also provides PVDF with intrinsic functional properties such as luminescent behavior and improved electrical performance, offering a simple and efficient strategy of nucleating specific PVDF phases.

## Graphical abstract

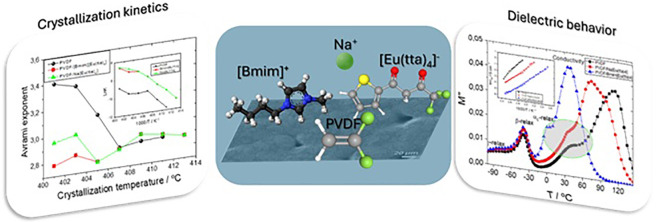

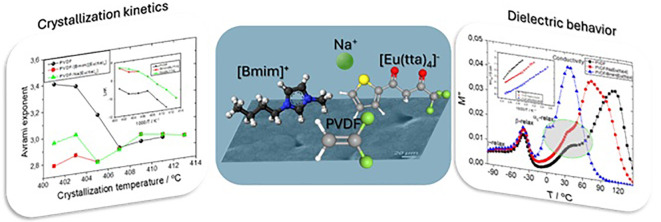

## 1 Introduction

Considering the Internet of Things (IoT) and Industry 4.0 paradigms, which allow the implementation of a more interconnected and interactive society, a new generation of innovative materials is needed to support the rapid advancement of technology in areas including sensors/actuators, biomedicine, and energy harvesting and storage.^1,2^ Smart and multifunctional materials are increasingly explored and used in this context due to their tailored responsiveness and processing versatility.^3,4^ Within this class of materials, hybrid ones based on high-performance polymers are increasingly explored, such as those composed of electroactive poly­(vinylidene fluoride), PVDF, considering their ferro-, pyro-, and piezoelectric properties.^5^ In particular, a new family of hybrid materials, resulting from the combination of PVDF and different functional ionic liquids (ILs), has garnered special attention in recent years.^6^ PVDF-IL hybrid materials are being investigated due to the combination of the PVDF properties—active response, chemical, mechanical, thermal, and radiation stability and simple processability—and the IL properties, i.e., chemical and electrochemical stability, nonflammability, ionic conductivity and negligible vapor pressure, together with a large range of tailorable functional responses, including ionic conductivity, magnetic, luminescent, and chromic responses, among others.^7–9^ Thus, different crystalline phases of PVDF, functional responses, and device integration can be achieved.^10,11^ PVDF is known for its polymorphism with four crystalline phases: α, β, γ, and δ, the crystallization phase depending on crystallization temperature and time. For example, when the polymer crystallizes by cooling from the melt at a reasonable rate, the nonpolar α-phase is produced. The material crystallizes in two coexisting phases when crystallization is carried out at temperatures higher than 155 °C, and during longer crystallization times α and γ-PVDF is produced.^5^ As the crystallization temperature increases, the amount of γ-PVDF present in the sample also increases. β-PVDF is promoted by casting from a polar solvent solution, through crystallization temperatures below 70 °C, and by the addition of different fillers when the polymer is processed from melt.^5^ This is the case of IL addition, which affects the crystallization kinetics and the crystalline phase, leading to the nucleation of the β- and γ-phases of PVDF.^11^

The CF_2_ bonds of PVDF and imidazolium type IL anchored to substrates by biomimetic catecholic attachment chemistry provide the ion–dipolar interactions that allow the PVDF polymorphs to be explored.^12^ The same occurs via direct blending of different IL types with PVDF, the specific ion–dipole interactions defining phase type and content within the polymer matrix.^5^

For crystallization from high temperatures (210 °C), the addition of [Emim]-based ILs leads to the crystallization of PVDF in α-phase and β-phase, the β-phase varying between 14 and 95% depending on the IL type, where 1-ethyl-3-methylimidazolium chloride [Emim]­[Cl] is the most effective IL for increasing β-phase content.^11^ The PVDF crystallization with the ionic liquid (IL) 1-ethyl-3-methylimidazolium hexafluorophosphate ([Emim]­[PF_6_]) has been monitored from isothermal crystallization at different temperatures, the addition of the IL inducing PVDF crystallites in the β and γ electroactive phases.^13^ Furthermore, 1-hexyl-3-methylimidazolium chloride ([HMIM]­Cl) was found to promote the formation of β-phase PVDF during the recrystallization process.^14^ The crystallization kinetics of PVDF with 1-butyl-3-methylimidazolium hexafluorophosphate ([BMIM]­[PF_6_]) was studied by two thermal steps: α-phase crystallization and further annealing at a higher temperature. The transformed lamellar morphology is dependent on the crystallization temperature of the α-phase.^15^

Furthermore, the PVDF crystallization with 1-hexadecyl-3-methylimidazolium bromide [C_16_mim]­[Br] demonstrated that the inclusion of 1 wt % of [C_16_mim]­[Br] in the PVDF matrix accelerates the crystallization of PVDF in the (β/γ) crystal form.^16^

PVDF crystallization from dimethylformamide (DMF) and dimethyl sulfoxide (DMSO) solutions at 100 °C and 80 °C with 1-butyl-3-methylimidazolium nitrate, [Bmim]­[NO_3_], and 1-tetradecyl-3-methylimidazolium nitrate, [Tdmim]­[NO_3_] promotes the formation of γ and β phases.^17^

Also, PVDF crystallization with phosphonium and imidazolium-based ILs (1 wt %) was studied, where ILs based on phosphonium cation combined with phosphinate anion exhibit strong interaction with fluoride chains, modifying the crystalline structure of the PVDF polymer. This effect increases the crystallization of the polar γ-phase and is observed in the presence of phosphonium-based ILs.^18^

Thus, the crystallization behavior of PVDF blends with the IL depends on the choice of anion and cation and the corresponding amount.

In the framework of the development of the next generation of functional PVDF-hybrid materials, herein, we study and demonstrate the effect of the cation type of ILs based on the [Eu­(tta)_4_]^−^ ion on the PVDF crystallization process. The rationale for choosing this anion was the luminescent properties displayed by this Eu complex anion. Two ILs have been investigated: sodium tetra­(2-thenoyltrifluoroacetonate)­europate­(III) Na­[Eu­(tta)_4_] and 1-butyl-3-methylimidazolium tetra­(2-thenoyltrifluoroacetonate)­europate­(III), [Bmim]­[Eu­(tta)_4_].^19^

PVDF blends with Na­[Eu­(tta)_4_] and [Bmim]­[Eu­(tta)_4_] (20 wt %) were prepared by solvent casting. The crystallization kinetics of the PVDF matrix within these hybrid systems were investigated using Fourier transform infrared spectroscopy (FTIR) and differential scanning calorimetry (DSC) at various temperatures to evaluate the formation of electroactive phases. Isothermal crystallization experiments were further analyzed by DSC using the Avrami formalism to gain insight into the nucleation and growth mechanisms. Additionally, dielectric relaxation spectroscopy was performed to explore the molecules mobility, relaxation processes and conductivity properties associated with the polymer matrix and the embedded luminescent centers. This approach contributes to the scientific understanding of hybrid materials with luminescent properties, highlighting their potential for advanced applications.

## 2 Experimental Section

### 2.1 Materials

PVDF (6010) with a molecular weight between 300–330 kDa and N,N-dimethylformamide (DMF) (99.5% purity) were supplied by Solvay and Merck, respectively. The Na­[Eu­(tta)_4_] (*M*_w_ = 1060.65 g/mol) and [Bmim]­[Eu­(tta)_4_] (*M*_w_ = 1176.88 g/mol) IL were synthesized according to the procedure reported in detail in ref. 19. The chemical structures of the cation [Bmim]^+^ and anion [Eu­(tta)_4_]^−^ are shown in Scheme 1. The ionic and hydrodynamic radius of the [Na]^+^ and [Bmim]^+^ cations are equal to 0.102 and 0.127 nm at 25 °C, respectively.^20^ For these species, the [Na]^+^ and [Bmim]^+^ volume are 0.004 nm^3^ and 0.15–0.18 nm^3^, respectively.

**Scheme 1:**
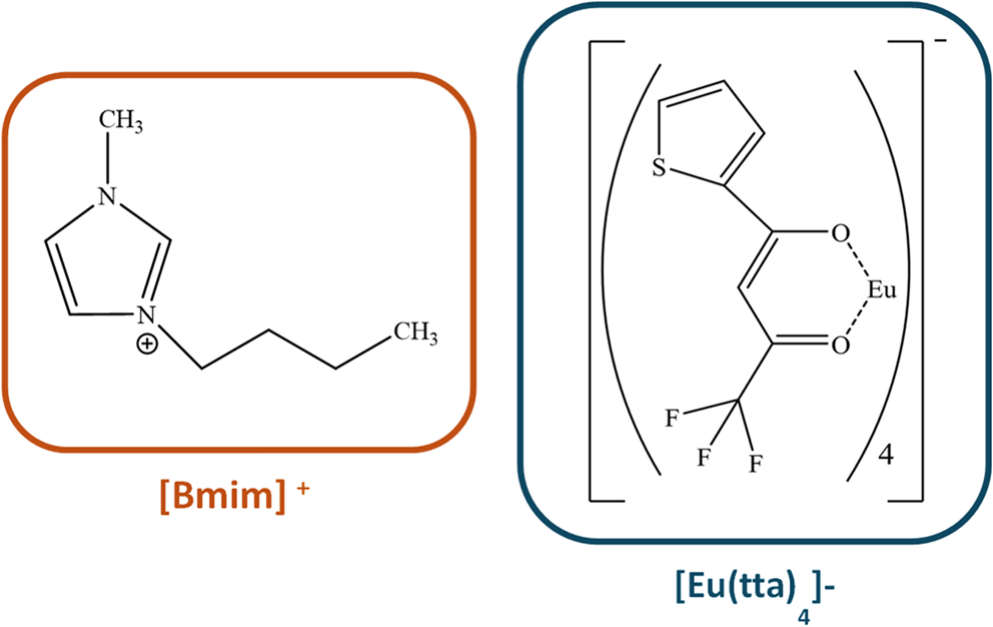
Chemical Structure of [Bmim]­[Eu­(tta)_4_]­^a^

a The anion is the same for Na­[Eu­(tta)_4_].

### 2.2 Sample Preparation

PVDF, PVDF/Na­[Eu­(tta)_4_], and PVDF/[Bmim]­[Eu­(tta)_4_] films were produced by the solvent casting method using DMF as the solvent, following the procedure reported in ref. 21. Initially, the equal amounts Na­[Eu­(tta)_4_] and [Bmim]­[Eu­(tta)_4_] (20% w/w) was dispersed in DMF separately for 15 min. This specific ILs and their content are selected as they provide a suitable functional response while still maintaining appropriate film-forming characteristics. After this process, PVDF was dissolved in each solution using the same polymer/solvent ratio (15/85% w/w). Additionally, a PVDF/DMF solution was also produced. After complete dissolution of PVDF in these solutions, each was spread out onto a glass substrate and oven-dried at 210 °C for 10 min in a standard oven (P-Selecta), ensuring total solvent removal.^21^ All films were produced with a final thickness of ∼44 μm.

### 2.3 Characterization Techniques

#### 2.3.1 Film Characterization

A Zeiss Ultra 55 instrument was used for field-emission scanning electron microscopy (FESEM) at 2 kV with an in-lens type II secondary electron detector. Using a Bal-Tek SCD005 system, samples were gold sputtered for 90 s in an argon environment to improve imaging resolution and surface conductivity.

FTIR spectra were acquired in the attenuated total reflection (ATR) mode using a Spectrum 100, PerkinElmer, in the range of 4000 to 400 cm^–1^ with a resolution of 4 cm^–1^. DSC measurements were performed using a PerkinElmer DSC 8000 under N_2_ atm (flow rate: 20 mL/min). Two sets of samples (5–10 mg) were analyzed using different thermal protocols. The first set aimed to explore thermal behavior at subambient temperatures. This protocol consisted of heating from −80 to 40 °C, cooling from 40 °C to −80 °C, and a second heating from −80 to 40 °C, with a scan rate of 20 °C/min.

The second set of samples was analyzed to characterize the crystallization process and determine the crystallinity. This protocol involved heating to 200, cooling to 0 °C, and subsequent heating to 200 °C at a scan rate of 20 °C/min.

To determine the sample’s crystallinity degree, eq 1 was used:^22,23^

1 $$X_{c}\left(\%\right)=\frac{{\Delta H}_{s}}{{x\Delta H}_{\alpha }+{y\Delta H}_{\beta }+{z\Delta H}_{\gamma }}\times 100$$

in which *X*_c_(%) corresponds to the sample’s crystallinity degree expressed in percentage, *x*, *y*, and *z* to the respective fractions of each crystalline phase. Δ*H*_s_, Δ*H*_α_, Δ*H*_β_, and Δ*H*_γ_ correspond to the melting enthalpies of the sample and pure crystals of α, β, and γ structures, with values of 93.07, 103.40, and 105.60 J/g, respectively.^22,23^

Dielectric relaxation spectroscopy (DRS) experiments were performed using a high-resolution α-S impedance analyzer (Novocontrol Technologies). Temperature control was achieved by a Novotherm system (Quatro Cryosystem), which uses a heated nitrogen gas stream derived from liquid nitrogen. Samples were placed between gold-plated parallel electrodes in a capacitor configuration.

Isothermal dielectric spectra were recorded at 5 °C intervals over a temperature range from −100 to 150 °C. The frequency domain extended from 0.1 to 10^6^ Hz. Data analysis was carried out through the equivalent complex properties permittivity, ε* (*f*), and electrical modulus, M*­(*f*), as they are interrelated through the following equations

2 $$M^{*}\left(\omega \right)=1/\varepsilon^{*}\left(\omega \right)=M^{\prime }\left(\omega \right)+iM^{\prime \prime }\left(\omega \right)=\frac{\varepsilon^{\prime }\left(\omega \right)}{\varepsilon^{\prime 2}\left(\omega \right)+\varepsilon^{\prime \prime 2}\left(\omega \right)}+i\frac{\varepsilon^{\prime \prime }\left(\omega \right)}{\varepsilon^{\prime 2}\left(\omega \right)+\varepsilon^{\prime \prime 2}\left(\omega \right)}$$

where ε′ and ε″ represent the real and imaginary components of the complex permittivity and ω = 2π*f*.

Additionally, the real conductivity, σ′(*f*), was also analyzed.

#### 2.3.2 Crystallization Characterization

With a diamond crystal in the horizontal micro-ATR Golden Gate unit (SPECAC), KBr beam splitter, deuterated triglycine sulfate (DTGS) detector, and Nicolet 6700 spectrometer, the crystallization kinetics was obtained from Fourier transform infrared spectroscopy (FTIR) spectra in the attenuated reflection mode with 64 scans.

A small amount of the sample was applied to the diamond crystal at room temperature. It was then heated to 200 °C and maintained in a molten condition for 5 min. After that, it was cooled to the desired crystallization temperature, ranging between 152 and 136 °C, at a rate of 15 °C/min rate. The ATR-FTIR spectral collection was initiated upon reaching the desired crystallization temperature. At each isothermal crystallization period, sixty-four images with a spectral resolution of 4 cm^–1^ were coadded to obtain a satisfactory signal-to-noise ratio.

Equation 3 was used to determine the electroactive (EA) phase fraction (sum of β and γ) of PVDF:^24–27^

3 $$F\left(\mathrm{EA}\right)=\frac{A_{\mathrm{EA}}}{\left(\frac{K_{\mathrm{EA}}}{K_{a}}\right)A_{a}+A_{\mathrm{EA}}}$$

where the percentage of the EA phase is represented by *F*(EA), the deconvoluted peak areas at 766 and 840 cm^–1^ (corresponding to α and EA phases, respectively) are identified by *A*_a_ and *A*_EA_ and their corresponding absorption coefficients, namely, 6.1 × 10^4^ and 7.7 × 10^4^ cm/mol, by *K*_a_ and *K*_EA_.^24,28^

Equation 4 was used to separate the independent contributions of β and γ bands from the overall EA phase

4 $$F\left(\gamma \right)=F\left(\mathrm{EA}\right)\times \left(\frac{A_{\gamma }}{{A_{\gamma }+A}_{\beta }}\right)\times 100$$

where the percentage of the γ-phase is represented by *F*(γ), the overall percentage of the electroactive phase by F­(EA), and the relative intensity absorbance at 1234 and 1275 cm^–1^ (corresponding to β and γ peaks, respectively) by *A*_β_ and *A*_γ_.^29^

Additionally, isothermal crystallization assessments were performed. Samples were heated to 200 °C, held for five min, and then cooled at the highest cooling rate (120 °C/min) to reach the selected crystallization temperatures (128 to 160 °C, in 2 °C increments). This procedure ensured that the DSC remained under control throughout the entire cooling scan. Avrami’s equation was applied to isothermal crystallization thermograms following eqs 5 and 6:^30–32^

5 $$\frac{X_{ct}}{X_{c\infty \left(T\right)}}=1-\exp\left(-kt^{n}\right)$$

and

6 $$\mathrm{q̇}\left(t\right)=X_{c\infty }\left(T\right)\frac{\rho_{c}}{\rho }\Delta H_{f}^{0}\frac{dX_{c}}{\mathrm{dt}}$$

where *X*_c_(*t*) is the crystalline fraction at time *t*, *X*_c_∞(*T*) is the maximum crystalline fraction at the crystallization temperature *T*, *k* is the overall crystallization rate constant, *n* is the Avrami exponent, *q̇*(*t*) is the DSC heat flow of the sample at time *t*, ρ, and ρ_c_ are the densities of semicrystalline PVDF and the crystalline phase, respectively, and Δ*H*_f_^0^ is the melting enthalpy of a perfect crystal.

## 3 Results and Discussion

### 3.1 Samples Crystallized on Cooling: Morphology, Polymer Phase, and Thermal Analysis

The morphological effect of the presence of the ILs in the PVDF polymer is illustrated in the FESEM images (Figure 1a–c). In these figures, typical PVDF spherulites are observed. The inclusion of the IL does not affect the presence of the spherulites but it affects their quantity and shape.^10,13^ In Figure 1b, the presence of IL on the sample surface can be identified by dark gray lines following the spherulite contours. The IL acts as a nucleation agent, as evidenced by the smaller and more densely packed spherulites compared with neat PVDF (Figure 1a), indicating that more spherulites grow simultaneously and collide during crystallization. Regarding the Na­[Eu­(tta)_4_] salt (Figure 1c), several stains are visible on the sample surface, which are attributed to physical segregation between the ILs and PVDF. SEM images indicate that the presence of ILs significantly affects PVDF morphology. The differences observed between Figure 1b,c can be attributed to specific interactions between the IL cations and the polymer chains.

**Figure 1:**
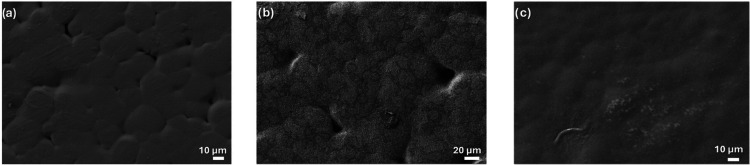
FESEM surface images of (a) neat PVDF, (b) PVDF/[Bmim]­[Eu­(tta)_4_], and (c) PVDF/Na­[Eu­(tta)_4_].

The FTIR spectra of the samples are shown in Figure 2, where the characteristic vibration bands of the species and the PVDF polymer are represented. The characteristic vibration bands corresponding to the [Eu­(tta)_4_] anion are indicated by arrows in Figure 2. The band at 1600 cm^–1^ corresponds to the C=O vibration, at 1550 cm^–1^ to C=C and C_4_H_3_S and the one at 1300 cm^–1^ is related to the C–F3 vibration.^33,34^ The vibration bands of the PVDF polymer are identified by the vertical lines of the α and β phases of PVDF, and it is shown that the addition of the ILs does not affect these vibration bands. The characteristic bands of the β phase at 840 and 1232 cm^–1^ and those of the α phase at 765, 796, and 976 cm^–1^ are detected.^11^

**Figure 2:**
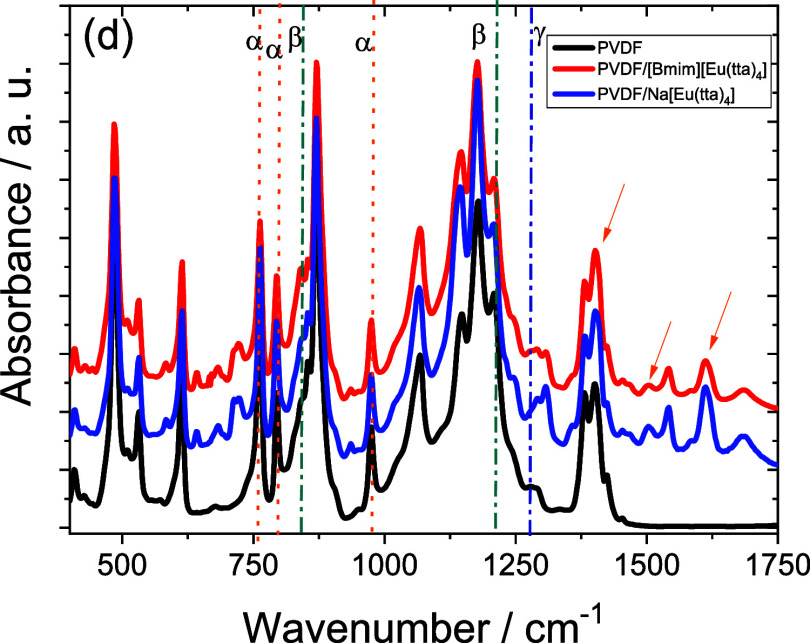
FTIR spectra of the different samples.

The crystalline fractions of the EA phase (β and γ) as well as the α phase, obtained from eqs 3 and 4, are included in Table 1. The electroactive fraction increases from 30% in neat PVDF to 37% in PVDF/[Bmim]­[Eu­(tta)_4_], while it remains essentially unchanged (≈ 29%) for PVDF/Na­[Eu­(tta)_4_]. It has been reported that, upon incorporation of IL into PVDF matrices, and independently of the IL type, an increase in the intensity of the β phase characteristics absorption bands is observed, consistent with crystallization into the all-trans (TTT) planar zigzag conformation of the PVDF chain. ILs act as β-phase nucleating agents through electrostatic interactions between the PVDF chain with the positive/negative charges of the IL.^35,36^ In the PVDF/[Bmim]­[Eu­(tta)_4_] blend, the imidazolium cation engages the negatively charged CF_2_ dipoles, while the IL anion interacts with positively polarized CH_2_ segments, establishing electroactive β structures.^36^ By contrast, the PVDF/Na­[Eu­(tta)_4_] blend exhibits an EA fraction comparable to neat PVDF, in line with ionic salts acting predominantly as heterogeneous nucleants that may increase overall crystallinity but do not necessarily toward β/γ phases.^37^

**Table 1:** Relative Content (%) of the Crystalline Phases (α, EA) of Neat PVDF and Blends with 20% IL Content, and Thermal Parameters Obtained from DSC

	PVDF	PVDF/[Bmim][Eu(tta)_4_]	PVDF/Na[Eu(tta)_4_]
*F*(α)%	70	63	71
*F*(β)%	19	23	16
*F*(γ)%	11	14	13
*T*_m_/°C	171	175	169
Δ*H*_s_/J·g^‑1^	51	72	69
*X*_c_/%	53	59	57
*T*_c_/°C	137	134	133

The effect of the IL type on the thermal properties of PVDF was analyzed from DSC scans. Figure 3a shows the DSC heating scan for all samples, where endothermic peaks related to the melting behavior of the PVDF crystalline phases are observed. A complex peak is observed due to the presence of different crystalline phases and the type of cation present. The thermal behavior parameters obtained in Figure 3 are presented in Table 1.

**Figure 3:**
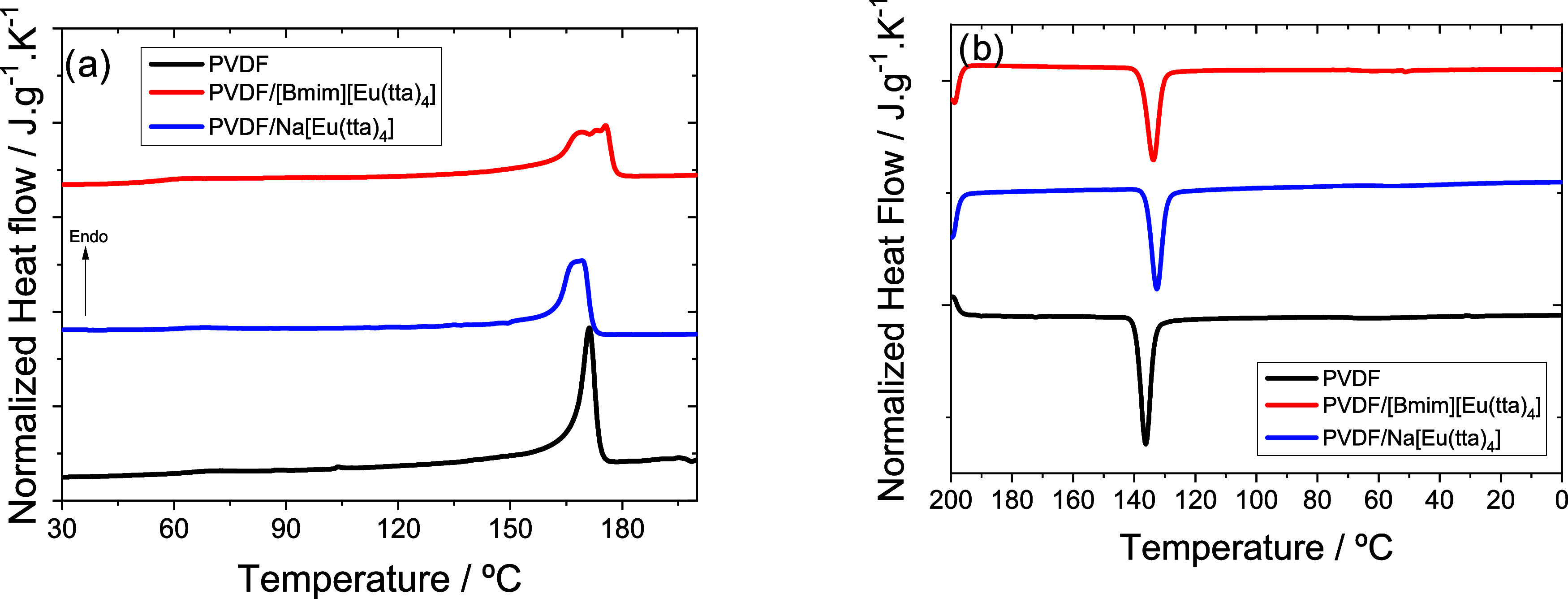
DSC scans of neat PVDF, PVDF/[Bmim]­[Eu­(tta)_4_], and PVDF/Na­[Eu­(tta)_4_] blends: (a) heating and (b) cooling.

The typical double melting peak of PVDF is obtained for the pure sample (Figure 3a) and is associated with melting occurring in two events. The addition of the IL influences the PVDF’s crystallization. The biggest difference is observed between the presence/absence of IL and not so much when the two ILs are compared. Differences between ILs are associated with the cation effect, Na^+^ or [Bmim]^+^, since both ILs share the same anion.

Neat PVDF displays a single melting peak at 171 °C, as commonly reported.^11^ The composite samples with ILs are characterized by broader peaks, including a lower temperature shoulder. This broad melting transition in the composites is consistent with a more heterogeneous crystalline population, coexisting lamellae of different thickness, and thermal stability. Overall crystallinity was also affected by the presence of ILs, with a slight increase in overall crystallinity after their incorporation. Among the cations, Bmim had a stronger effect in promoting crystallization. This modest change is consistent with literature reports where ionic liquids and inorganic salts can act as nucleating agents, promoting crystal formation and enhancing the crystalline fraction.^11,37^

Figure 3b presents the cooling thermograms, featuring an exothermic peak associated with crystallization that shifts slightly to lower temperatures in the blends, consistent with modest plasticizing effects of the ionic species (IL or salt).^10,38^ The addition of the ILs shifts this peak to higher temperatures, likely due to the increase in the number of crystallization nuclei already detected in the microscopy images. Crystallization temperatures for the study of the isothermal crystallization kinetics were selected based on the thermal behavior observed in Figure 3.

### 3.2 Isothermal Crystallization Kinetics

#### 3.2.1 Crystalline Phase Contents

The crystallization rate and the crystalline phases that form during isothermal crystallization depend significantly on temperature. In order to make the study of the crystallization process in blends with ionic liquids comparable to that of pure PVDF, the minimum exothermic temperature measured in the cooling scan, which we have called *T*_c_ in Table 1, was taken as a reference. FTIR spectrum measurements were taken at different times during the crystallization process at *T*_c_, *T*_c_+4, and *T*_c_+8 °C in the three systems studied.

The tests were carried out by rapidly cooling the sample inside the equipment to the temperature at which isothermal crystallization took place and acquiring the spectrum at different points in time during the crystallization process. Figure 4 shows the FTIR spectra of all samples at the moment when the isothermal crystallization temperature is reached (which we call time 0) and the spectrum taken at the longest crystallization time, 3700 s. The complete set of intermediate points is available as Supporting Information Figure S1. It can be seen in Figure 4a that, in crystallization at 146 °C, PVDF already shows the presence of the characteristic peaks of the α phase at 765, 796, and 976 cm^–1^ in the first spectrum taken at time 0, which then grow in intensity as the crystallization process progresses at that temperature. At 138 °C (Figure 4b), the intensity of these peaks at time 0 is lower. At the end of the isothermal crystallization process, the spectrum closely resembles that found at 148 °C, but quantitative analysis will reveal significant differences as shown below.

**Figure 4:**
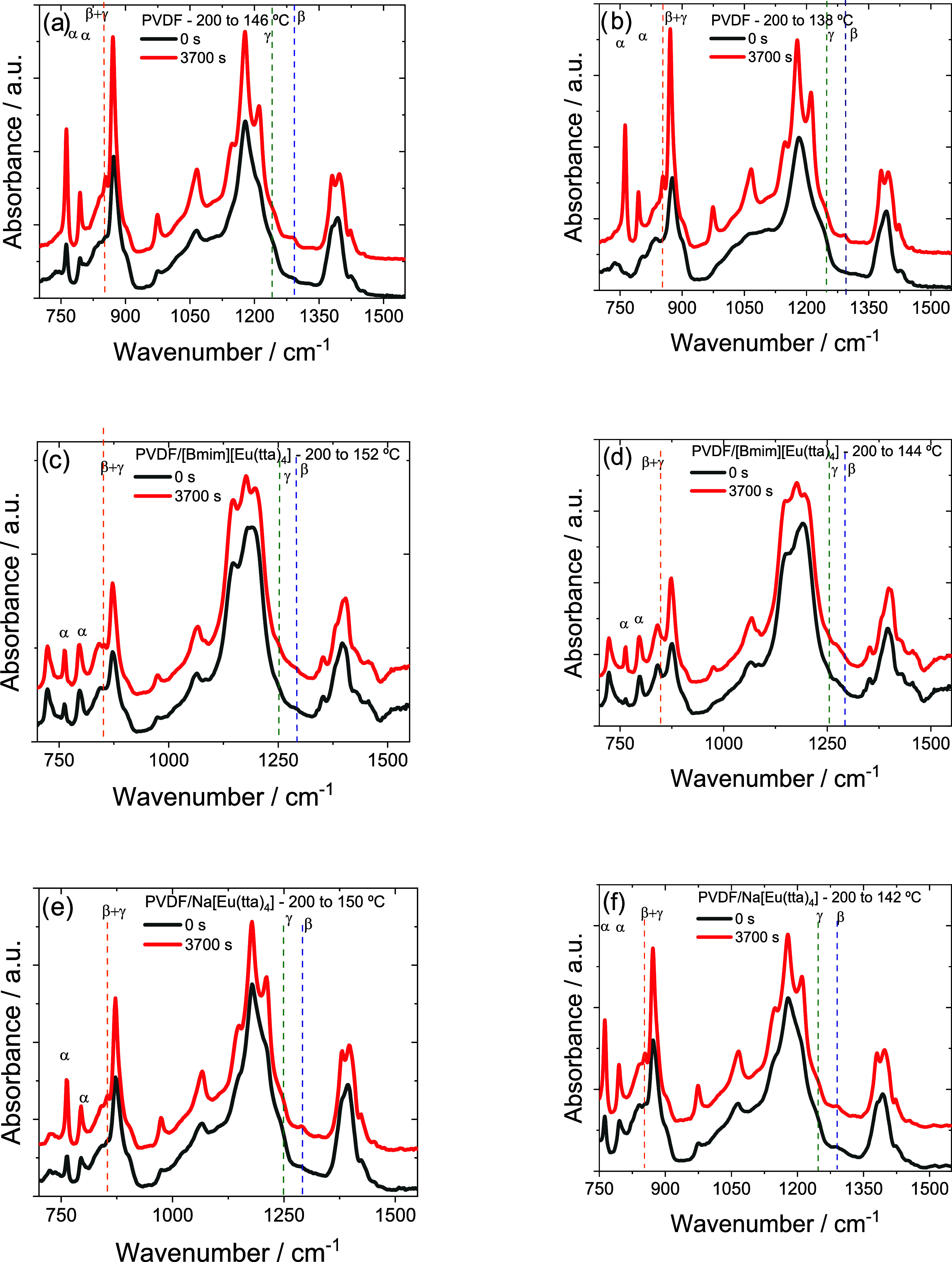
FTIR absorbance spectra of samples crystallized for 0 and 3700 s (bottom to top). Neat PVDF from 200 °C to (a) 146 and (b) 138 °C, PVDF/[Bmim]­[Eu­(tta)_4_] from 200 °C to (c) 152 and (d) 144 °C, and PVDF/Na­[Eu­(tta)_4_] from 200 °C to (e) 150 and (f) 142 °C.

The crystallization of the PVDF/[Bmim]­[Eu­(tta)_4_] compound shows the appearance of the 840 cm^–1^ peak characteristics of electroactive phases from the start of the isothermal crystallization process and the growth of its intensity during isothermal crystallization. The peaks of the α phase are also shown. It is curious that the peak at 765 cm^–1^ is formed during isothermal crystallization, while the peak at 796 cm^–1^ already appears at time 0 (Figure 4c,d). The spectra of PVDF/Na­[Eu­(tta)_4_] behave in a manner that is somewhat intermediate between the two previous ones because the ability of Na­[Eu­(tta)_4_] to induce the formation of electroactive phases is lower than that of [Bmim]­[Eu­(tta)_4_], as we see below.

The quantitative analysis is based on eqs 1 and 2. The relative intensities of the characteristic peaks of the different phases were calculated from deconvolution of the spectrum in the intervals between 750 and 850 cm^–1^, as shown in Figure 5 for one of the crystallization temperatures of each compound. The procedure is analogous for the other crystallization temperatures. The deconvolution to quantify the relative presence of β and γ phases was performed between 1200 and 1300 cm^–1^ (Supporting Information Figure S1). Table 2 shows the amount of the crystalline phase formed at different temperatures.

**Figure 5:**
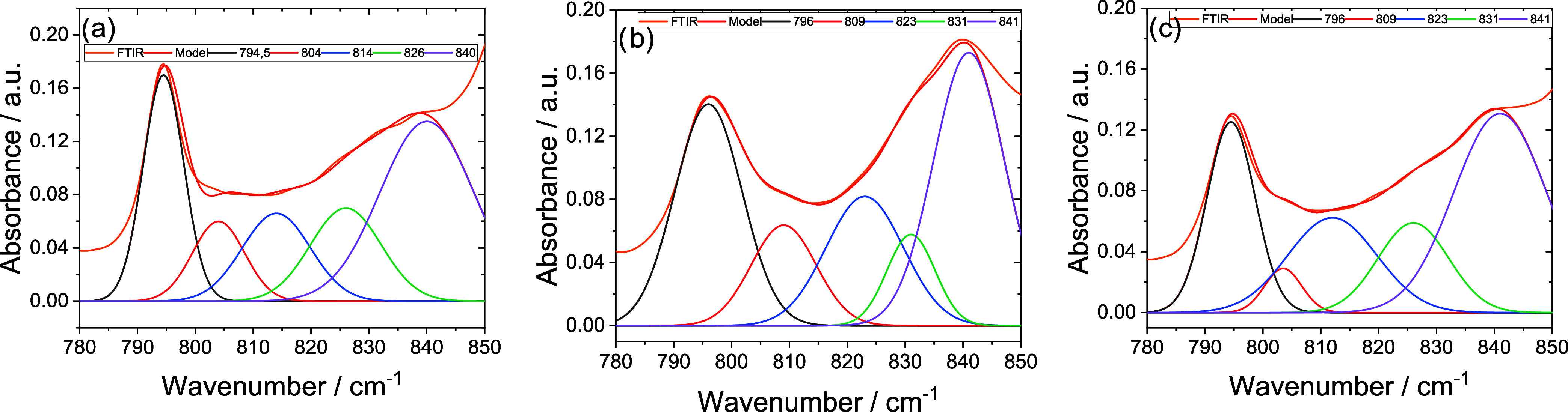
Results of the deconvolution of the FTIR spectra between 780 and 850 cm^–1^ for (a) PVDF crystallized at 150 °C, (b) PVDF/[Bmim]­[Eu­(tta)_4_] crystallized at 144 °C, and (c) PVDF/Na­[Eu­(tta)_4_] crystallized at 146 °C.

**Table 2:** Percentages of α, β, and γ Relative Crystalline Phases after Crystallization at Different Temperatures (*T*_c_)­^a^

sample	PVDF			PVDF/[Bmim][Eu(tta)_4_]			PVDF/Na[Eu(tta)_4_]		
*T*_c_ (°C)	138	142	146	144	148	152	142	146	150
*F*(EA)	24	24	24	57	52	48	33	34	35
*F*(α)%	76	76	76	43	48	52	68	66	65
*F*(β)%	7	8	8	27	25	20	11	12	13
*F*(γ)%	17	17	16	29	27	28	21	22	22

a Error of 2%.

To quantify the crystalline structures, the method presented in ref. 26 was applied, based on eqs 2 and 3. Figure 5 shows the results of the deconvolution of the spectra between 780 and 850 cm^–1^ for samples crystallized at specific temperatures. For other temperatures, this procedure was applied similarly.

Table 2 shows the amount of crystalline phase crystallized at different temperatures, demonstrating that the different IL, differing in the cation, affects the final relative phase content of the samples.

Neat PVDF predominantly crystallizes in the α-phase, regardless of the crystallization temperature. The 840 cm^–1^ band, to which both the β and γ phases contribute, can also be identified. The electroactive fraction is mainly composed of the γ-phase.

IL [Bmim]­[Eu­(tta)_4_] is a potent inducer of crystallization in the electroactive phases, which at the lowest crystallization temperatures account for 57% of the total crystalline phases. Compared with pure PVDF, the PVDF/[Bmim]­[Eu­(tta)_4_] system shows a particularly large increase in the β-phase fraction. In contrast, the increase is quite small in the PVDF/Na­[Eu­(tta)_4_] system. The difference between the behavior of these systems must be associated with the strong interaction of the Bmim cation with the PVDF chain segments, while the interaction of the Na+ ion has a lesser effect.

#### 3.2.2 Crystallization Kinetics

The isothermal DSC thermograms of PVDF/[Bmim]­[Eu­(tta)_4_] crystallized at various temperatures are shown in Figure 6a. The exothermic peak changes to higher times, and the peak width increases as the crystallization temperature (*T*_c_) rises, as seen in Figure 6a. This is because when *T*_c_ rises, the rate of crystallization decreases. Additionally, each sample demonstrates this attribute.

**Figure 6:**
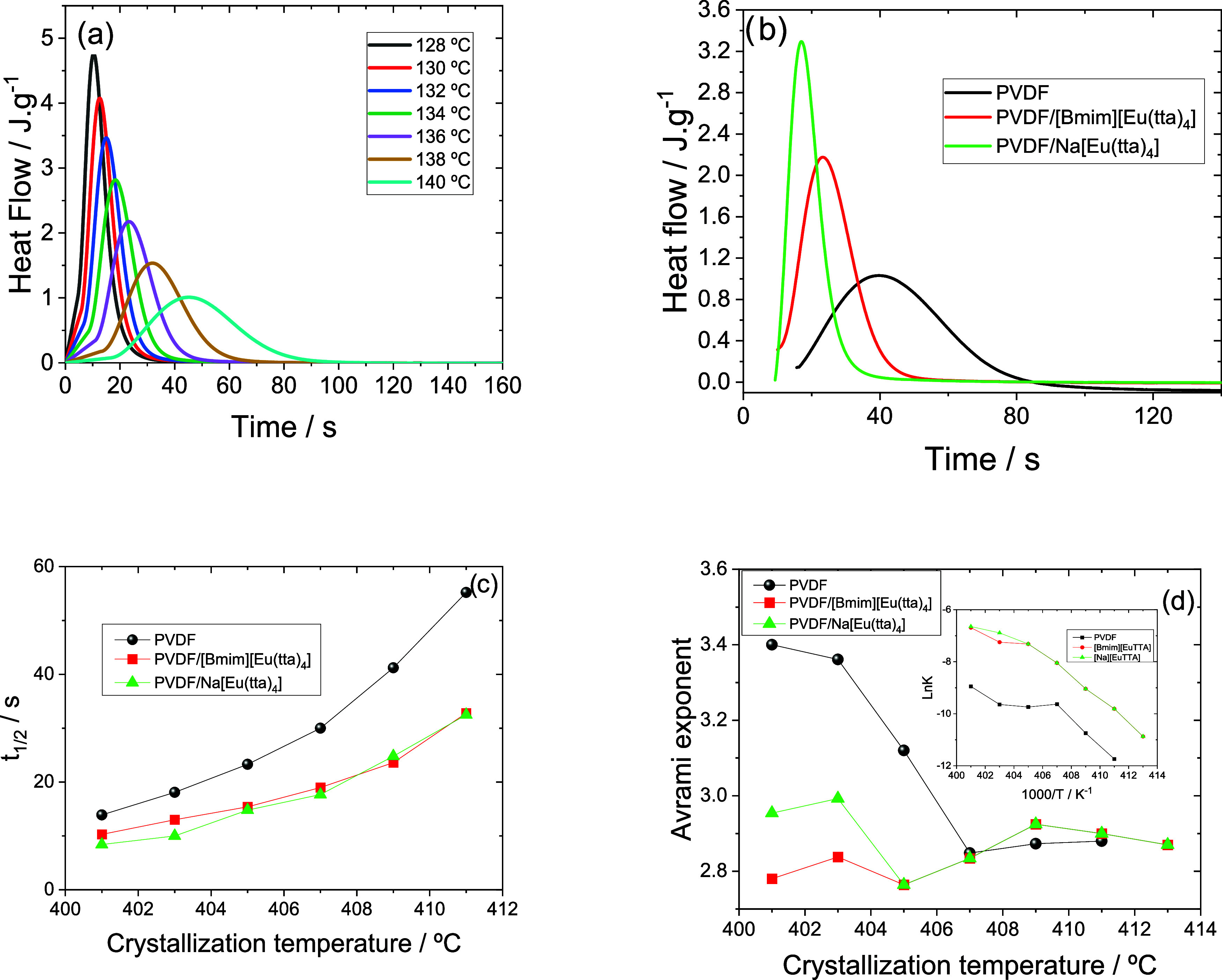
(a) Isothermal crystallization of the PVDF/[Bmim]­[Eu­(tta)_4_]; (b) DSC isotherms for the crystallization at 136 °C for all samples; (c) *t*_1/2_ and (d) Avrami’s exponent (*n*) as a function of crystallization temperature for all samples (inset: LnK vs *T*_c_).

The DSC isotherms for crystallization at 136 °C are shown in Figure 6b for all samples. It is observed that, in comparison to neat PVDF, the peak corresponding to the highest crystallization rate is significantly displaced to lower times for the samples filled. According to the results of crystallization on cooling (Figure 3b), this behavior indicates that the IL content accelerates the PVDF crystallization kinetics. The half-time crystallization (*t*_1/2_), which is shown in Figure 6c at all crystallization temperatures illustrates this characteristic. The Avrami exponent (*n*) and LnK for each sample, obtained after fitting with eq 4, are shown as a function of the crystallization temperature in Figure 6d. From Figure 6c, the Avrami exponent depends on the crystallization temperatures and IL content. For lower crystallization temperatures, this value is less than 3 and depends on the type of cation, being related to the different crystalline phases that form in the samples. For crystallization temperatures above 406 K, the value is about 3 for all samples and crystallization temperatures.

#### 3.2.3 Melting

Isothermal crystallization tests in the DSC allow for a broader sweep of crystallization temperatures and characterization of the kinetics of the process, but the effect of the fraction of the different crystalline phases formed on the thermal response is moderate. It is known that the different α, β, and γ phases have slightly different melting temperatures, but on the other hand, the temperature at which the crystals form also has a major influence on the melting temperature. Thus, it has been reported that the γ phase in PVDF has a higher temperature than the α and β phases, but as the γ phase forms at higher temperatures than the others, the result found is actually a combination of the effect of the crystallization temperature and that of the crystalline structure itself.

In our crystallization tests, after the isothermal process at a temperature *T*_c_, a heating sweep is performed during which the crystallites that formed at *T*_c_ melt. During the sweep interval, the sample is always above the temperatures that would allow recrystallization, which often occurs in DSC sweeps of samples crystallized at low temperatures or crystallized during cooling. Thus, in the results shown in Figure 7, the appearance of double peaks can be more reliably correlated to the presence of two different crystalline phases.

**Figure 7:**
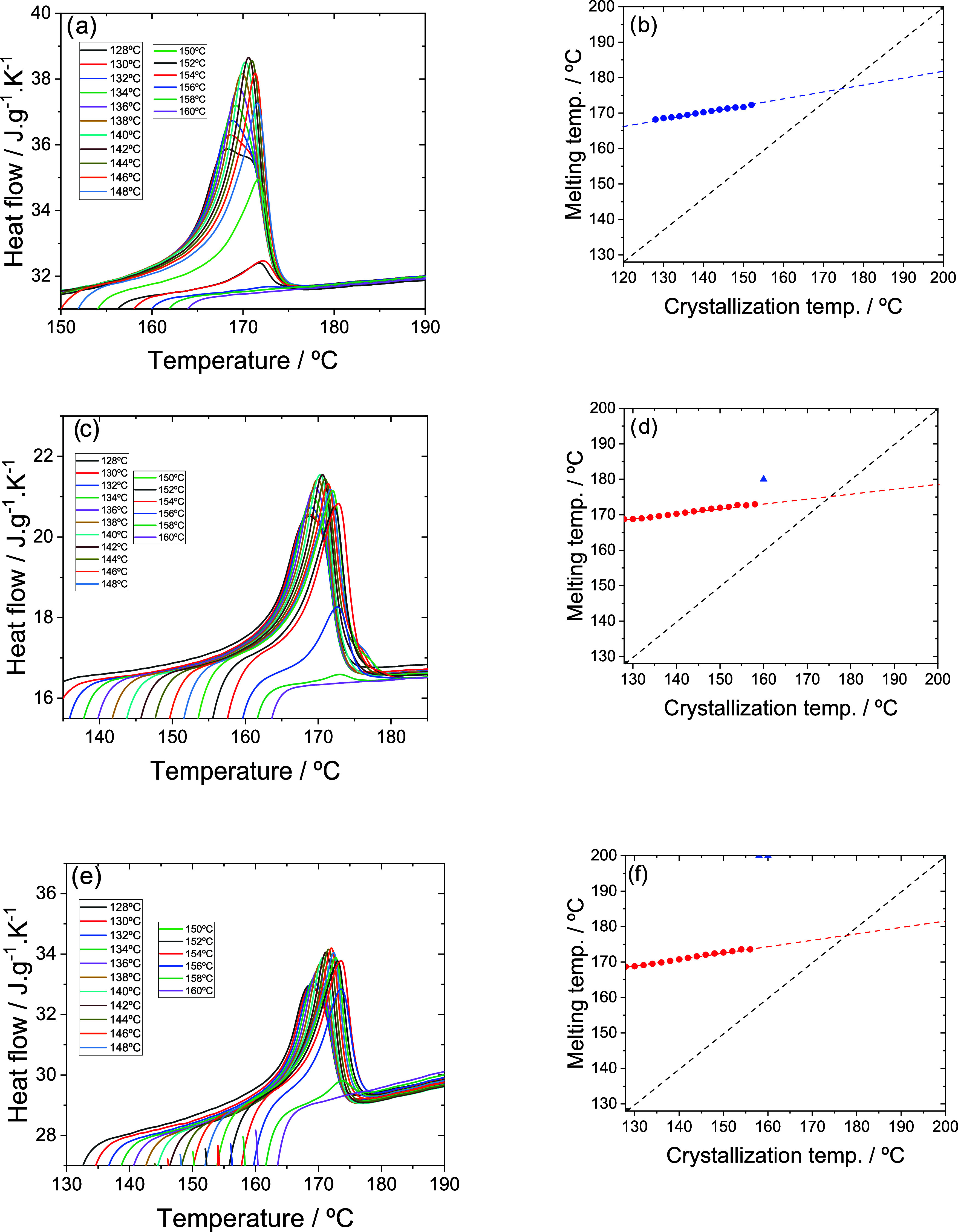
DSC thermograms and the melting temperature (*T*_m_) (the maximum of the endothermic peak) as a function of the isothermal crystallization temperature (*T*_iso_) for (a) and (b) neat PVDF, (c) and (d) PVDF/[Bmim]­[Eu­(tta)_4_] and (e) and (f) PVDF/Na­[Eu­(tta)_4_], respectively. The gray line in (b–f) represents *T*_m_ = *T*_iso_.

Figure 7a clearly shows the effect of crystallization temperature *T*_c_ on the melting temperature *T*_m_ (the temperature of the endothermic peak maximum) and the appearance of double peaks. At low crystallization temperatures, up to *T*_c_ = 136 °C, it appears that the endothermic peak is the result of the superposition of two peaks; however, at higher crystallization temperatures, melting produces a single narrow melting peak. The thickness of the crystalline lamellae increases with *T*_c_; consequently the melting temperature also increases. When *T*_m_ is plotted against *T*_c_ (Hoffman–Weeks diagram), a linear relationship is observed that would extrapolate to an equilibrium melting temperature, where *T*_m_ = *T*_c_ = 176 °C.

In the compounds, a change in shape of the melting peaks is observed compared to pure PVDF (Figure 7c,e), with a single peak generally present except in the PVDF/ [Bmim]­[Eu­(tta)_4_] system. For *T*_c_ > 150 °C, this system shows a shoulder on the high-temperature side of the melting peak, which can be attributed to the melting of γ crystals. This change in shape does not translate into a significant change in the slope of the Hoffman–Weeks diagram (Figure 7c,f).^39^

An interesting fact is that in pure PVDF, the intensity of the melting peak drops rapidly from the crystallization temperature of 148 °C (Figure 7a) and a few degrees above this, the polymer practically does not crystallize. In contrast, in compounds with any of the two ILs, the melting peak height remains close to its maximum value up to 154 °C (Figure 7b,c). This effect may be associated with greater γ-phase crystallization in compounds with ILs, which would form at higher temperatures.

### 3.3 Dielectric Behavior

#### 3.3.1 Dielectric Permittivity and Electric Modulus Formalism

The effect of the cation type on dielectric behavior was investigated using dielectric spectroscopy. The imaginary component of the complex permittivity (ε’’) is shown in Figure 8. Figure 8a–c illustrates ε’’ as a function of frequency for all samples at different temperatures. In all cases, ε’’ decreases with increasing frequency, which is attributed to polarization mechanisms.^40^ At a given frequency, the imaginary part of the dielectric permittivity increases with increasing temperature, due to the enhanced molecular mobility and electrical conductivity induced by thermal activation.^41^

**Figure 8:**
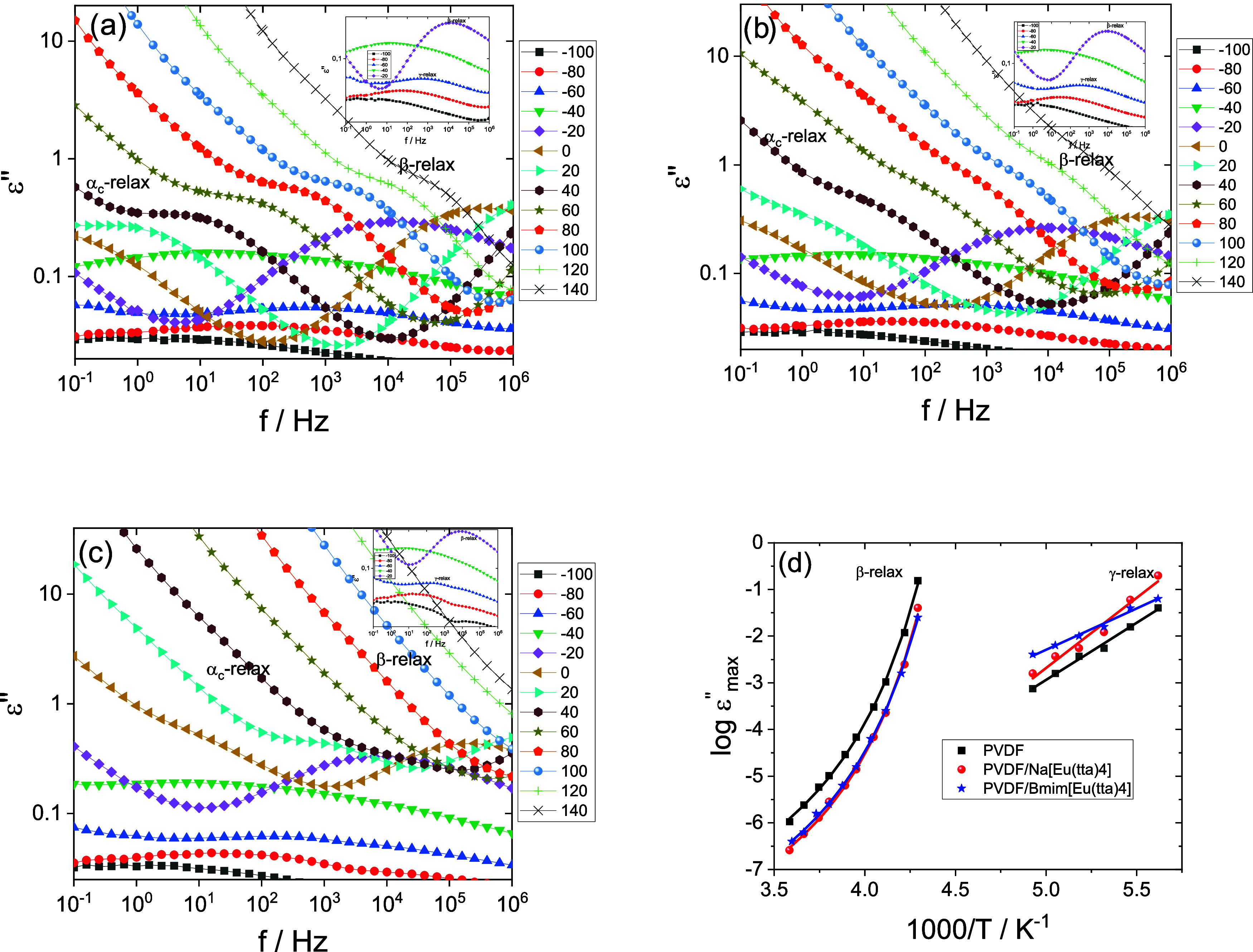
Frequency dependence of ε’’ for PVDF and PVDF/IL blends at various temperatures. (a) Neat PVDF; (b) PVDF/Na­[Eu­(tta)_4_]; (c) PVDF/[Bmim]­[Eu­(tta)_4_]. Solid lines are included as visual guides. The inset highlights the low-temperature range in greater detail. (d) Temperature dependence of the relaxation times obtained from the maxima of ε’’, corresponding to the structural β-relaxation and the secondary γ-relaxation of the PVDF matrix. Solid lines in (d) represent Vogel–Fulcher–Tammann (VFT) fitting for the β-relaxation and Arrhenius fitting for the γ-relaxation.

For the same frequency, the imaginary part of the dielectric permittivity increases with increasing temperature due to its effect on molecular mobility and electrical conductivity.^41^

Three relaxation processes are found in Figure 8a–c, associated with the polymer matrix: the structural β-relaxation, which in low-molecular-weight glass formers is called the α-process and is associated with the cooperative motions underlying the glass transition, the secondary relaxation γ-relaxation, and a third process α_c_-relaxation that can be attributed to the mobility at the interface between the crystalline and amorphous phases.^42^ This last process appears in all samples, irrespective of the incorporation of the IL, and its analysis is hindered by the overlapping contributions of conductivity and a possible Maxwell–Wagner–Sillars (MWS) effect.^43,44^ The latter can develop at the interfaces between distinct dielectric media with different constituents or phases, as observed in semicrystalline polymers,^45^ including PVDF composites^46^ or even in boundaries between grains in crystalline materials.^47^

The β and γ-relaxations of the samples were analyzed in detail, as shown in Figure 8d.

A Vogel–Fulcher–Tammann (VFT) law was used to simulate the temperature dependence of the relaxation times of the β-relaxation:^48^

7 $$\tau_{\beta -\mathrm{relax}}\left(T\right)=\tau_{0}e^{B/T-T_{0}}$$

where τ_0_ represents the relaxation time at infinite temperature, *B* is a material-dependent constant, and *T*_0_ is the Vogel temperature, below which molecular rearrangements become kinetically frozen.

On the other hand, an Arrhenius law was fitted to the temperature dependence of the relaxation times of the γ-relaxation (secondary relaxation at temperatures below *T*_g_):^49^

8 $$\tau_{\gamma -\mathrm{relax}}\left(T\right)=Ae^{-E_{a}/K_{B}T}$$

where *A* is a pre-exponential factor, *E*_a_ is the apparent activation energy associated with the relaxation process, and *K*_B_ is the Boltzmann constant.

The fitting parameters obtained in the VFT and Arrhenius models for both relaxations are listed in Table 3.

**Table 3:** VFT Fitting Parameters Obtained for the Structural β-Relaxation and Arrhenius Fitting Parameters Obtained for the Secondary γ-Relaxation of PVDF and PVDF/IL Blends

	β-relaxation [−40 to −5 °C]				γ-relaxation [−95 to −70 °C]	
	τ_0_ (s)	*B* (K)	*T*_0_ (K)	*T*_g‑diel_ (K) (τ = 100 s)	τ_0_ (s)	*E*_a_ (kJ/mol)
PVDF	5.55 × 10^–10^	591	202	225	6.89 × 10^–16^	47
PVDF/Na[Eu(tta)_4_]	1.79 × 10^–10^	560	203	224	1.96 × 10^–18^	58
PVDF/[Bmim][Eu(tta)_4_]	2.12 × 10^–10^	575	202	223	7.04 × 10^–12^	34

Table 3 shows that the cation type does not significantly affect the β-relaxation process but affects the γ-relaxation. The activation energy of this relaxation is higher for the larger cation ([Bmim]^+^).

As represented in Figure 9, the complex electric modulus (M*) formalism allows for enhancing the resolution of the relaxation peaks related to charge transport and molecular dynamics within the bulk material.

**Figure 9:**
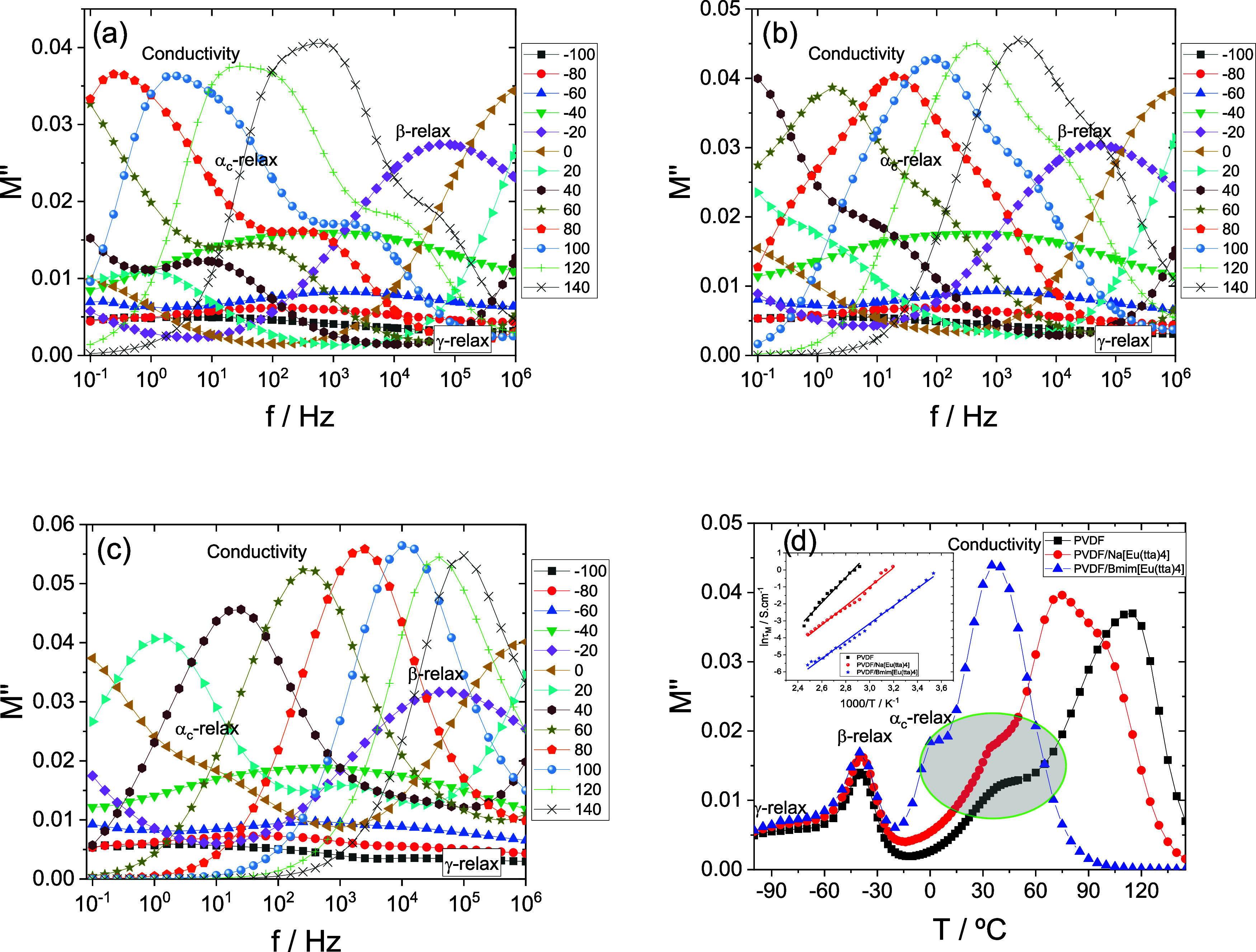
Frequency dependence of *M*’’ for PVDF and PVDF/IL blends at various temperatures. (a) Neat PVDF; (b) PVDF/Na­[Eu­(tta)_4_]; (c) PVDF/[Bmim]­[Eu­(tta)_4_]. Solid lines are included as visual guides; (d) Isochronal curves of *M*’’ at 10 Hz as a function of temperature. The inset in (d) shows the temperature dependence of the relaxation times associated with the conductivity process, obtained from the maxima of *M*’’ in the isothermal curves. Solid lines represent the Arrhenius fitting of the conductivity process.

Figure 9a–c shows the imaginary part of the complex electric modulus (*M’’*) for all samples in the temperature range from −100 to 140 °C where the different relaxation processes are identified, together with the electrical conductivity behavior.

From the analysis of Figure 9a–c, it can be seen that the incorporation of the ionic liquid (IL) does not affect the β-relaxation, which emerges centered around −35 °C in the isochronal plot at 10 Hz, irrespective of the material or the γ-relaxation of the polymer matrix compared to neat PVDF. However, it significantly influences the α_c_-relaxation and the conductivity behavior, as shown in detail in Figure 9d for a frequency of 10 Hz. This effect is likely related to interactions between the ILs and the interfacial regions between the crystalline and amorphous domains, which modify local chain mobility and relaxation dynamics.

The high-temperature contribution of conductivity to the modulus, *M*’’, does not manifest as a single, well-defined peak; rather, it exhibits a more complex behavior, particularly in the blend PVDF/Na­[Eu­(tta)_4_], Figure 9d. This multimodal response may be attributed to the above-mentioned Maxwell–Wagner–Sillars effect associated with the interfacial region separating distinct dielectric phases that can arise from (i) poor dispersion of the ionic liquid or (ii) distinct crystalline phases characterized by different dielectric constants.

As shown in Figure 9d, the incorporation of the IL shifts the onset of conductivity to lower temperatures, an effect more pronounced in PVDF/[Bmim]­[Eu­(tta)_4_], which can be attributed to differences in cation volume influencing charge transport and interfacial polarization mechanisms. This will be explored in the next section while analyzing the real conductivity.

An Arrhenius law was fitted to the temperature dependence of the maxima of the conductivity contribution to *M*’’

9 $$\tau_{M}\left(T\right)=\tau_{0}e^{{-E}_{a}/K_{B}T}$$

where τ_0_ is a pre-exponential factor, *E*_a_ is the apparent activation energy associated with the conductivity process, and *K*_B_ is the Boltzmann constant.

The Arrhenius fitting parameters for the conductivity peaks are listed in Table 4. As shown, the addition of the ILs (either the salt or the IL) enhances the conductivity behavior, and this enhancement is influenced by the type of cation. In particular, the blend containing the larger cation [Bmim]^+^ exhibits a lower activation energy than the one with Na^+^, indicating that the cation volume influences the energy barrier for ion transport within the PVDF matrix.

**Table 4:** Arrhenius Fitting Parameters Obtained from the Maximum of *M*’’ for the Conductivity Process of PVDF and PVDF/IL Blends

	*T* range (°C)	τ_0_ (s)	*E*_a_ (kJ/mol)
PVDF	[70,135]	8.64 × 10^22^	141
PVDF/Na[Eu(tta)_4_]	[40,130]	1.43 × 10^18^	108
PVDF/[Bmim][Eu(tta)_4_]	[10,130]	2.02 × 10^19^	99

#### 3.3.2 Complex Conductivity

Figure 10 shows the real component of the complex conductivity (σ’), resembling the behavior exhibited by a variety of semiconducting disordered materials.^50^ At high frequencies and low temperatures, conductivity is frequency-dependent (*ac* regime), converting to a frequency-independent behavior at low frequencies and high temperatures, a region where a plateau emerges (*dc* regime). While the *ac* response is due to a short-distance subdiffusive charge transport mechanism, *dc* conductivity originates from a long-distance diffusive charge migration regime.^51^

**Figure 10:**
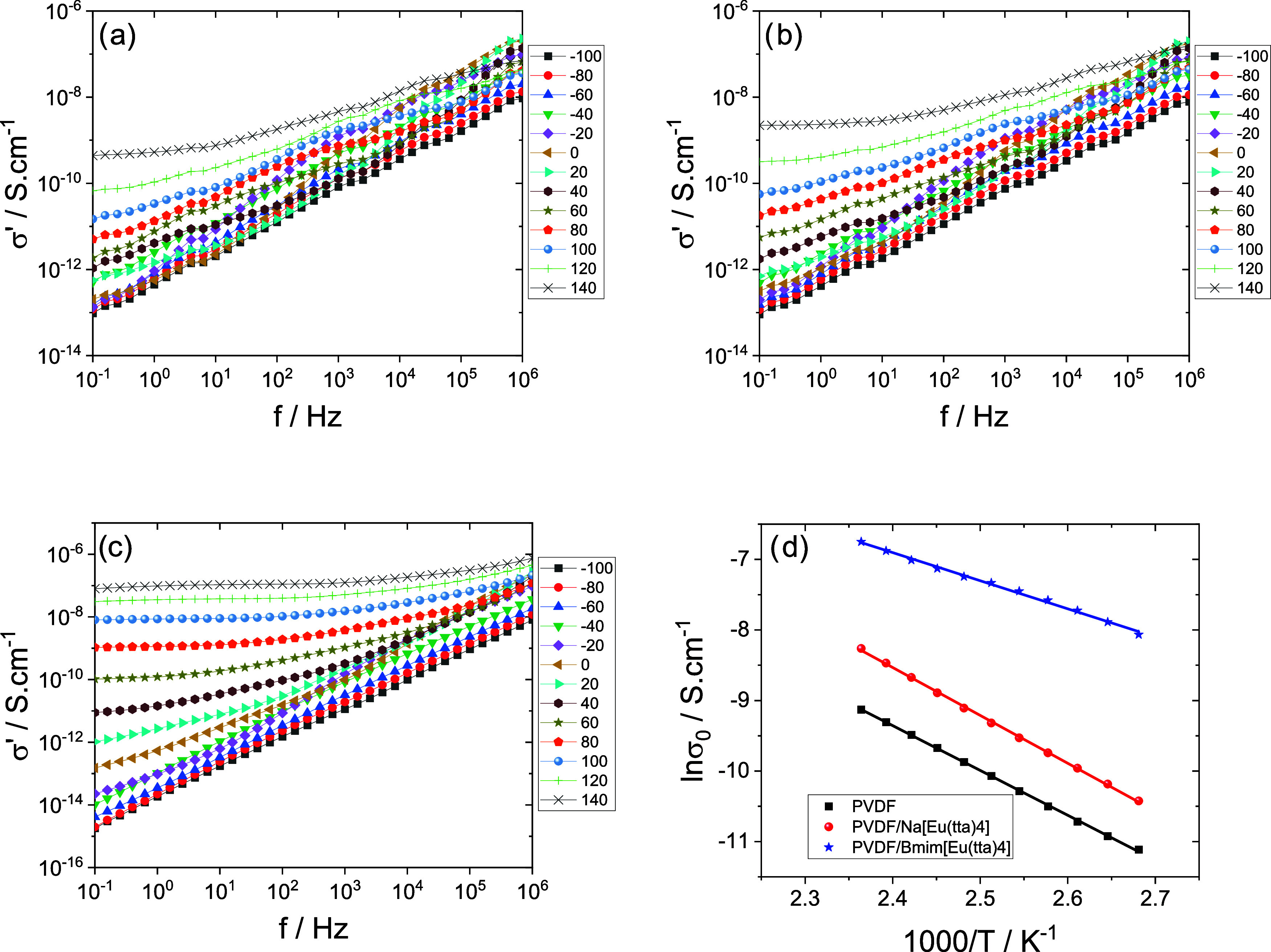
Frequency dependence of the real part of the conductivity (σ’) at different temperatures for PVDF/IL blends. (a) Neat PVDF, (b) PVDF/Na­[Eu­(tta)_4_], and (c) PVDF/[Bmim]­[Eu­(tta)_4_]. Solid lines are included as visual guides. (d) Temperature dependence of dc conductivity (σ_dc_) as a function of the different IL-containing samples. Solid lines represent the Arrhenius fitting.

The initial rise in σ’ at low frequencies, although not pronounced in this system, is attributed to the blocking of charge carriers at the electrode interfaces. This is followed by a plateau region (σ_0_), corresponding to dc conductivity, which is more evident in the PVDF blend with the larger cation [Bmim]^+^, and then by the frequency-dependent increase in ac conductivity.

Figure 10a shows that the real part of the conductivity, σ’(ω), increases with frequency for all temperatures. Further, there is a plateau region that is more noticeable at higher temperatures and is related to dc conductivity contributions.

The conductivity values for the IL-containing samples (Figure 10b,c) increase significantly. There is a plateau that represents the dc conductivity (σ_0_), which is larger for ILs with larger cation volumes. This is followed by a linear increase at higher frequencies that is indicative of the ac conductivity.

Interestingly, in the IL-containing samples, conductivity increases more significantly for PVDF/Na­[Eu­(tta)_4_]. In the latter, the *dc*-plateau emerges at lower temperatures and extends, at the highest temperature, to almost all the frequency range, meaning that a percolative conduction regime was attained.^52^ Thus, the incorporation of the IL with the smaller cation (Na^+^) enhances long-range conduction, promoting percolation. This size effect appears to outweigh the morphological changes induced by IL incorporation. Indeed, the overall crystallinity decreases upon IL addition and is lower in the [Bmim]^+^-based blend than in the Na^+^-based one. If the matrix morphology were the dominant factor governing conductivity, then a higher conductivity would be expected for the [Bmim]^+^-based blend; however, the opposite trend is observed. Therefore, the results suggest that the cation volume plays a more decisive role in determining the conductivity behavior, which opens doors for future applications of the PVDF/Na­[Eu­(tta)_4_] blend.

Among the two, the PVDF/[Bmim]­[Eu­(tta)_4_] sample exhibits the highest conductivity, highlighting the influence of the cation volume and mobility on ionic transport. Figure 9d presents the temperature dependence of dc conductivity, further illustrating the enhanced values observed in the sample containing the IL.

The fitting of σ_0_ was carried out using the Arrhenius model

10 $$\sigma_{0}\left(T\right)=\sigma_{\infty }e^{-E_{a}/K_{B}T}$$

where σ_∞_ is the conductivity extrapolated to infinite temperature, *E*_a_ is the apparent activation energy associated with the dc conductivity process, and *K*_B_ is the Boltzmann constant.

The Arrhenius fitting parameters for the dc conductivity (σ_0_) are presented in Table 5.

**Table 5:** Arrhenius Fitting Parameters Obtained for dc Conductivity (σ_0_) of PVDF and PVDF/IL Blends

	*T* range (°C)	σ_*∞*_ (S/cm)	*E*_a_ (kJ/mol)
PVDF	[100,150]	7.93 × 10^5^	122
PVDF/Na[Eu(tta)_4_]	[100,150]	5.71 × 10^7^	130
PVDF/Bmim[Eu(tta)_4_]	[100,150]	4.58 × 10^2^	76

Table 5 illustrates how the addition of IL increases the electrical conductivity response, which is dependent on the kind of cation and has a lower activation energy for the bigger cation.

By taking into account the time–temperature superposition principle,^53^ a single master curve could be obtained using the isotherms σ′ normalized with respect to σ_0_ and to the characteristic frequency (ω_c_), which is the junction of the ac conductivity straight line with the horizontal plateau in the log­(σ′) vs log­(ω) plot and the point at which the real part of the conductivity starts to increase with frequency. The value of ω_c_ for each temperature is more precisely determined by considering it as a fitting parameter in master curve creation. However, because this extrapolation is highly unpredictable, the characteristic frequency (ω_c_) is defined as the transition point between the low-frequency plateau, associated with dc conductivity, and the onset of ac conductivity. In pure ionic liquids, this frequency has been linked to the rate at which charge carriers overcome the energy barrier that hinders their transport.^54^

Figure 11a,b presents the master curves log­(σ’/σ_0_) vs log­(ω/ω_c_) for PVDF/Na­[Eu­(tta)_4_] and PVDF/[Bmim]­[Eu­(tta)_4_], respectively. Two distinct regions are observed: a low-frequency plateau corresponding to dc conductivity and a high-frequency regime indicative of ac conductivity. While the master curves demonstrate general scaling behavior, the overlap is not complete, indicating subtle differences in the temperature dependence of the conductivity process, likely due to variation in ion–polymer interactions or local structural effects.

**Figure 11:**
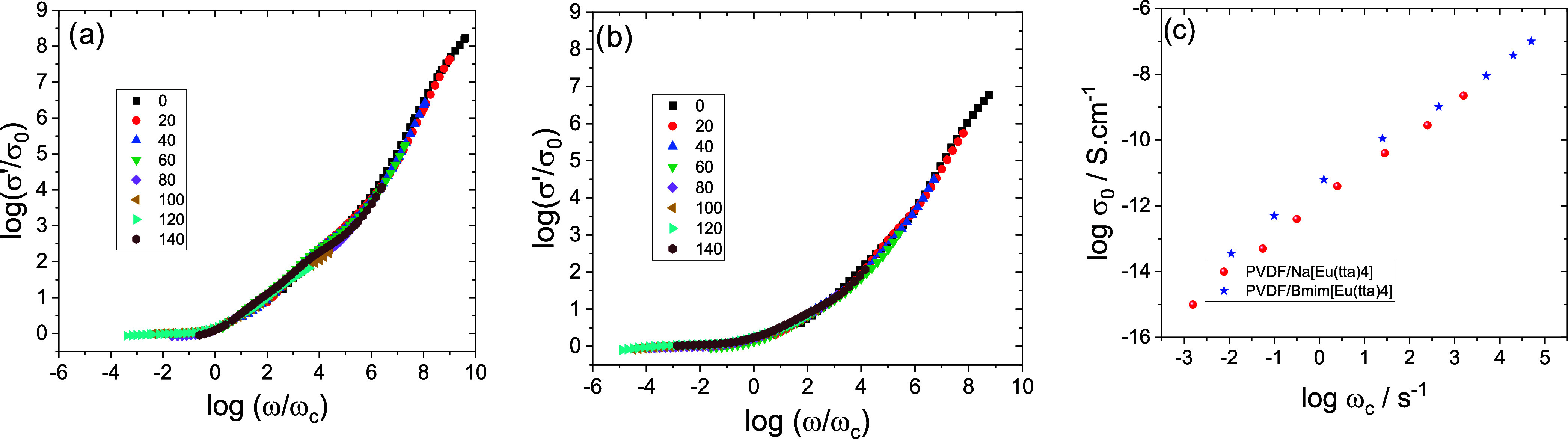
Scaling with respect to the characteristic frequency (ω_c_) and dc conductivity (σ_0_) at different temperatures. (a) PVDF/Na­[Eu­(tta)_4_]; (b) PVDF/[Bmim]­[Eu­(tta)_4_]; (c) temperature dependence of the characteristic frequency ω_M_ obtained from *M*″ and ω_c_; (dc) dc conductivity versus characteristic frequency.

Figure 11c depicts the relationship between ω_c_ and σ_0_ across several temperatures. A nearly linear correlation is observed in the log–log plot, consistent with the Barton-Namikawa-Nakajima (BNN) relation (σ_0_ ∼ ω_c_), which suggests similar mechanisms of charge transport in both blends.^55^ The PVDF/[Bmim]­[Eu­(tta)_4_] sample, however, exhibits significantly higher conductivity and lower activation energy, suggesting that while the mechanism remains comparable, the efficiency of ion transport is enhanced by the larger [Bmim]^+^ cation. This behavior aligns with previous results that cation structure has a limited impact on the transport mechanism but can influence the conductivity magnitude through mobility and polymer-ion interactions.^54^

## 4 Conclusions

PVDF blends incorporating the ionic liquid [Bmim]­[Eu­(tta)_4_] and salt Na­[Eu­(tta)_4_] were prepared to investigate the influence of the IL type and cation nature on polymer phase crystallization. FESEM images revealed that both ILs act as nucleation agents, significantly affecting the morphology of the PVDF matrix. The presence of the ILs promotes the formation of PVDF electroactive phases, with the extend of enhancement depending on the IL type within the crystallization temperature range of 128–160 °C. Notably, the [Bmim]­[Eu­(tta)_4_] blend exhibited a higher proportion of the electroactive β and γ phases compared to Na­[Eu­(tta)_4_] blend. At a given crystallization temperature, the γ phase exhibits a higher melting point than both the β and the α phases. Changes in the crystalline phase that forms and the nucleation effect of the ILs result in an increase in the crystallization temperature observed in cooling scans in the blends with respect to pure PVDF, contrary to what would be anticipated from the freezing point depression.

Dielectric analysis reveals three relaxation processes (β-relaxation, γ-relaxation, and α_c_-relaxation) associated with the polymer matrix. The structural β-relaxation was not significantly affected by the cation volume of the ionic liquids. The conductivity formalism demonstrated that samples containing larger cations exhibit higher dc conductivity and lower activation energy, confirming that the cation volume plays a key role in modulating ion transport behavior. The PVDF/[Bmim]­[Eu­(tta)_4_] sample showed the lowest activation energy for conduction, consistent with enhanced ionic mobility. These findings suggest that tailoring the electrical properties of PVDF-based materials through the careful selection of cation type offers a promising strategy for developing next-generation functional materials with specific behavior.

## Supplementary Material



## Notes

FTIR spectra crystallized at different temperatures (PDF)
