# Supplementary material for: Effect of Cation Type on the Isothermal Crystallization of Poly(vinylidene fluoride) Blended in Ionic Liquids with [Eu(tta)4]− Anion
Source: J Phys Chem C Nanomater Interfaces. 2026 Feb 20;130(9):3639–53. doi: 10.1021/acs.jpcc.6c00153 (PMC12969586; doi:10.1021/acs.jpcc.6c00153)
Supplement: Supplementary file 1 [file jp6c00153_si_001.pdf]

Supporting Information for Publication

Effect of Cation Type on the Isothermal Crystallization of Poly (vinylidene fluoride) Blended in Ionic Liquids with $[Eu(tta)_4]^-$ Anion

Luis A. Martins[1], José Luis Gómez Ribelles[1,2], Carlos M. Costa[3,4*], Verónica de Zea Bermudez[5], Daniela M. Correia[6], Madalena Dionisio[7], Andreu Andrio[8], Ivan Krakowsky[9], Roser Sabater i Serra[1,2], Senentxu Lanceros-Méndez[3,10,11], Isabel Tort-Ausina[1,2*]

[1]Centre for Biomaterials and Tissue Engineering, CBIT, Universitat Politècnica de València, C/Camino de Vera s/n, 46022 Valencia, Spain

[2]CIBER de Bioingeniería, Biomateriales y Nanomedicina, Instituto de Salud Carlos III.

[3]Physics Center of Minho and Porto Universities (CF-UM-UP) and Laboratory of Physics for Materials and Emergent Technologies, LapMET, University of Minho, 4710-057 Braga, Portugal

[4]Institute of Science and Innovation for Bio-Sustainability (IB-S), University of Minho, 4710-053 Braga, Portugal

[5]Chemistry Department and CQ-VR, University of Trás-os-Montes e Alto Douro, 5000-801 Vila Real, Portugal

[6]Centre of Chemistry, University of Minho, 4710-057 Braga, Portugal

[7]LAQV-REQUIMTE, Department of Chemistry, NOVA School of Science and Technology, Universidade Nova de Lisboa, 2829-516 Caparica, Portugal

[8]Departament de Física, Universitat Jaume I, 12071 Castelló, Spain

[9]Department of Macromolecular Physics, Charles University 180 00 Prague 8, Czech Republic

[10]BCMaterials, Basque Center for Materials, Applications and Nanostructures, UPV/EHU Science Park, 48940 Leioa, Spain.

[11]Ikerbasque, Basque Foundation for Science, 48009 Bilbao, Spain.

[*] Corresponding Authors

C.M. Costa (cmscosta@fisica.uminho.pt); Isabel Tort-Ausina (isatort@fis.upv.es)

S1 - FTIR spectra crystallized at different temperatures

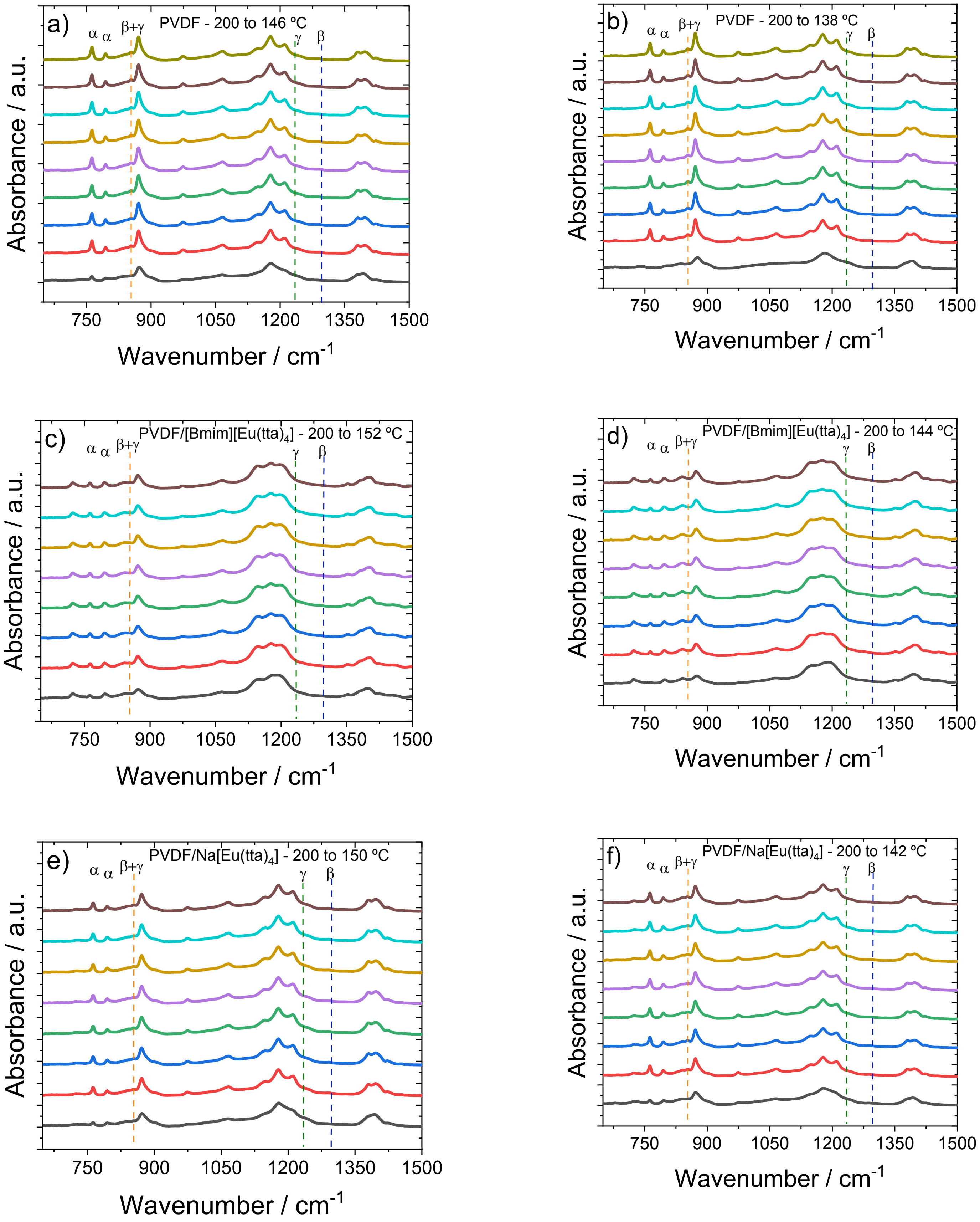

Figure S1- FTIR absorbance spectra of PVDF a) and b) and PVDF/ionic liquid blends c) to f) crystallized at specific temperatures for 0, 34, 74, 400, 700, 1300, 1900 and 3700 seconds (bottom to top)
